# Simple reverse genetics systems for Asian and African Zika viruses

**DOI:** 10.1038/srep39384

**Published:** 2016-12-19

**Authors:** Thérèse Atieh, Cécile Baronti, Xavier de Lamballerie, Antoine Nougairède

**Affiliations:** 1 UMR “Emergence des Pathologies Virales” (EPV: Aix-Marseille university - IRD 190 - Inserm 1207 - EHESP), Marseille, 13385, France; 2 Institut hospitalo-universitaire Méditerranée-Infection, Marseille, 13385, France

## Abstract

Zika virus (ZIKV), a typical example of a re‐emerging pathogen, recently caused large outbreaks in Pacific islands and the Americas, associated with congenital diseases and neurological complications. Deciphering the natural history, ecology and pathophysiology of this mosquito-borne pathogen requires effective reverse genetics tools. In the current study, using the bacterium-free ‘Infectious Subgenomic Amplicons’ (ISA) method, we generated and made available to the scientific community via the non-profit European Virus Archive collection, two simple and performing reverse genetics systems for ZIKV. One is based on an Asian ZIKV strain belonging to the outbreak lineage (French Polynesia 2013). The second was designed from the sequence of a low-passaged ZIKV African strain (Dakar 1984). Using the ISA procedure, we derived wild-type and a variety of specifically engineered ZIKVs in days (intra- and inter-lineage chimeras). Since they are based on low-passaged ZIKV strains, these engineered viruses provide ideal tools to study the effect of genetic changes observed in different evolutionary time-scales of ZIKV as well as pathophysiology of ZIKV infections.

Zika virus (ZIKV; *Flaviridae; Flavivirus*) is a small, enveloped, single-strand positive-sense RNA virus. Its genome of approximately 11 kb contains a unique open reading frame (ORF) and two flanking 5′ and 3′ noncoding regions. Once translated, the polyprotein is cleaved to release structural (capsid [C], premembrane/membrane [prM/M], and envelope [E]) and non-structural (NS1, NS2A, NS2B, NS3, NS4A, NS4B, and NS5) viral proteins^1^.

Human disease due to ZIKV infections remained relatively neglected until 2007, when a large ZIKV outbreak occurred in Micronesia^2^,^3^. ZIKV re-emerged in the Pacific Ocean islands in 2013, causing outbreaks in French Polynesia^4^, which was followed by a series of ZIKV outbreaks affecting numerous Pacific Ocean islands^5^,^6^. Recently, ZIKV invaded the Americas causing large outbreaks in many territories of South and Central America^7^,^8^,^9^,^10^. Prior to the French Polynesian outbreak, ZIKV human infections were described as mild and self-limiting. However, the recent outbreaks also revealed the existence of non-vector transmission of ZIKV via sexual or vertical transmission^11^,^12^,^13^,^14^ and recent studies revealed a strong association between ZIKV infection and a rise in detected cases of congenital malformations and neurological complications^15^,^16^.

ZIKV was primarily transmitted between sylvatic *Aedes* mosquitoes and non-human primates in the African forests. However, during the 1950 s the virus was sporadically isolated from peri-domestic *Aedes* mosquitoes and humans in Africa and subsequently in the 1960 s and 1970 s in both Africa and Asia^6^. Phylogenetic analysis of ZIKV genomic sequences revealed the existence of two distinct lineages (African and Asian) associated in the beginning with the geographical distribution of the virus. The viruses responsible for the current expansion to the Pacific Ocean islands, the Carribean and mainland Latin America form a unique monophyletic group (called outbreak lineage in the current study) descending from the Asian lineage^17^. The exact reasons why this viral pathogen suddenly invaded new territories and caused severe diseases remain unknown. One hypothesis, recently proposed by Pettersson *et al*., is that some mutations accumulated during the recent evolution of ZIKV are critical genetic determinants of change in the transmission and possibly pathogenicity phenotype of the virus^18^.

Reverse genetics systems, based on infectious clone technology, have been recently described for the Cambodian Asian ZIKV strain FSS13025^19^, the Brazilian Asian ZIKV strain Paraiba_01/2015^20^ and the MR766 protoype ZIKV strain^21^, a high passaged laboratory African strain. Stability of some of these infectious DNA clones^20^,^21^ was improved by inserting intron sequences in protein coding regions of the viral genome and the infectious clone based on the FSS13025 strain was modified by the introduction of a luciferase reporter gene^19^. Furthermore, the rapid bacterium-free reverse genetic method called ISA (infectious subgenomic-amplicons) that we developed few years ago^22^ was also used to generate wild-type and recombinant virus expressing the GFP reporter, based on the MR766 protoype ZIKV strain^23^. All these systems are valuable tools to study new antiviral compounds and the physiopathology of ZIKV infection.

Here, using the ISA procedure, we have generated two reverse genetics systems for ZIKV which are available for the scientific community via the European Virus Archive goes Global (EVAg) project, a non-profit organization (http://www.european-virus-archive.com/). One is based on a clinical isolate of Asian ZIKV belonging to the outbreak lineage. The second is based on a low-passaged African strain of ZIKV. By generating chimeric ZIKV strains (intra and inter-lineage chimeras), we demonstrate that this procedure conveniently allows the generation of genetically modified ZIKVs in days and thus will be a major tool for identifying genetic determinants associated with the recent expansion of the virus and its increased virulence.

## Results and Discussion

Two ZIKV strains with well-known laboratory passage histories were selected. The Asian strain PF is a clinical isolate derived from the serum of a patient infected in 2013 in French Polynesia and sequenced in our laboratory. The African strain DAK is a low-passaged strain isolated from an *Aedes taylori* mosquito in 1984 in Dakar, Senegal. For the latter virus, we used the GenBank sequence number KU955592 to design the reverse genetics system.

**Figure 1:**
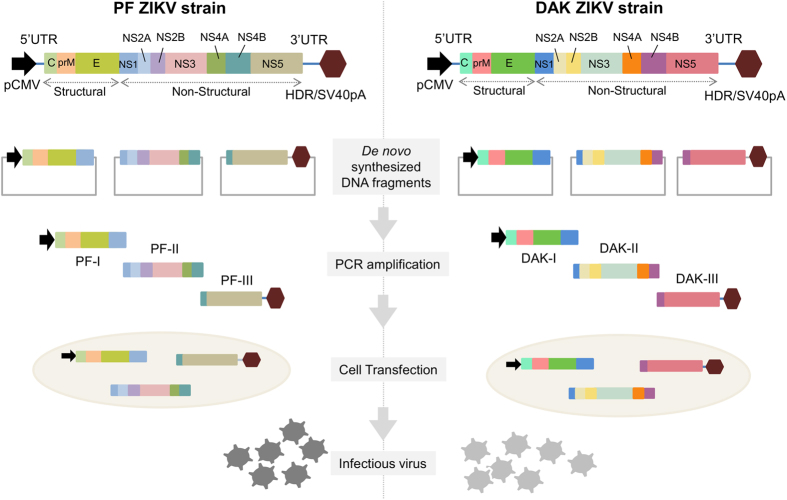
Schematic representation of the ISA method used to recover infectious ZIKVs. The entire viral genome, schematically represented in the figure, flanked respectively at the 5′ and 3′ untranslated regions by the pCMV and the HDR/SV40pA, was *de novo* synthesized in three double-stranded overlapping DNA fragments. Each of these was then amplified by PCR and all subgenomic products were pooled and transfected into permissive cells to generate infectious ZIKVs.

We used the previously described ISA procedure to implement both reverse genetics systems^22^. The schematic representation of the procedure used to recover infectious ZIKVs is presented in Fig. 1. Both complete ZIKV genomes flanked respectively at 5′ and 3′ termini by the human cytomegalovirus immediate early enhancer/promoter (pCMV) and the hepatitis delta ribozyme followed by the simian virus 40 polyadenylation signal (HDR/SV40pA) were *de novo* synthesized in three double-stranded DNA fragments of approximately 4.2, 4.3 and 3.5 kb that overlap by ≈70–80 pb. These synthetic genes were used as template to produce overlapping DNA fragments by PCR.

For each reverse genetics system, an equimolar mix of the three purified amplicons was used for cell transfection. Three different mammalian cell lines were used (BHK-21, SW13 and HEK-293 cells). Each of these ensures efficient replication of the parental PF strain with the production of a complete cytopathic effect (CPE). For amplification of the viruses, infectious cell supernatant media were then serially passaged twice using the same cell type.

Virus replication was demonstrated using a combination of several criteria as previously described^22^ (results summarized in Table 1): (i) detection of CPE, (ii) production of viral RNA in cell supernatant medium, (iii) production of infectious particles in cell supernatant medium, and (iv) verification of complete genome integrity.

1. From the first passage, a clear CPE was systematically observed in all experiments between days 4–7 post-inoculation except for DAK viruses in SW13 cells.
2. The production of viral genomes in cell supernatant medium was assessed using a real time RT-PCR assay at the second passage to ensure disappearance of the DNA used during the transfection. Average amounts of viral RNA detected ranged between 6.42 and 7.92 log_10_ copies per mL for PF viruses and between 6.74 and 7.43 log_10_ copies per mL for DAK viruses.
3. The production of infectious particles in cell supernatant medium was assessed at the second passage using a standard TCID_50_ assay. Average infectious titres ranged between 3.94 and 4.14 log_10_ TCID50/mL for PF viruses and between 5.02 and 5.19 log_10_ TCID50/mL for DAK viruses.
4. Verification of the complete genome sequence was performed at the second passage (only one replicate per condition) as previously described^24^. For each cell supernatant medium analyzed, NGS complete genomic sequencing confirmed the integrity of the genome structure and the genetic similarity (≥99.95%).

**Figure 2:**
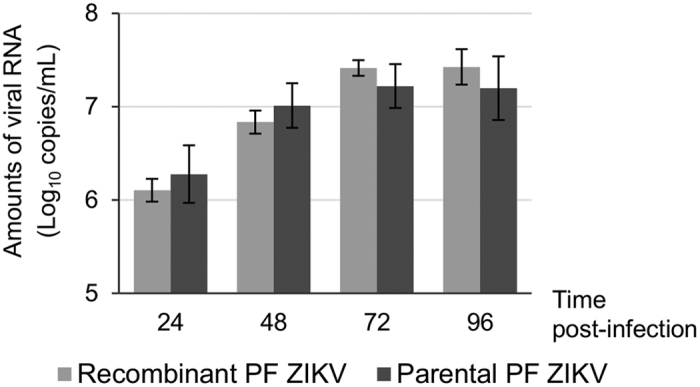
Virus replication kinetics with recombinant and parental PF ZIKV strains. An moi of 0.01 was used to infect BHK-21 cells with recombinant or parental PF ZIKVs produced in BHK-21 cells. Cells were washed and cell supernatant media were harvested at 24, 48, 72 and 96 hours post-infection and then analyzed using a real-time RT-PCR assay.

We compared *in cellulo* the recombinant PF virus with the parental PF viral strain. Replication kinetics in BHK21 cells of both viruses was characterised. Cell supernatant media were harvested at 24, 48, 72 and 96 hours post-infection. Amounts of viral RNA were measured using a real-time RT-PCR assay. Replication kinetics of parental and recombinant viruses were essentially similar (Fig. 2).

**Figure 3:**
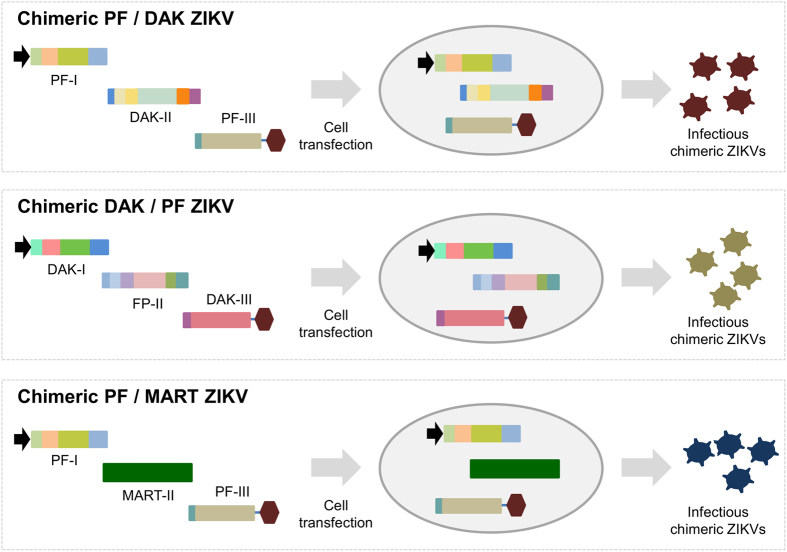
Rapid generation of chimeric ZIKVs using the ISA method. By simply exchanging DNA fragments, inter- and intra-lineage chimeric ZIKVs were produced in days using the ISA method. The MART-II DNA fragment was generated by RT-PCR from the cell culture supernatant medium of the MART ZIKV strain.

The versatility of both reverse genetics systems was further examined as follows (Fig. 3). We were able to produce two inter-lineage chimeric viruses in days by simply exchanging the second DNA fragment of PF and DAK ZIKVs. We also explored the possibility of inserting genomic fragments of other clinical strains of ZIKV into the PF strain. Accordingly, we used the cell culture supernatant medium of the MART ZIKV strain, an Asian strain isolated from a patient in Martinique in 2015, to produce the second fragment by RT-PCR. Once again, we were able to produce this inter-lineage chimeric virus in days by mixing this DNA fragment with the first and the third DNA fragment of the PF ZIKV strain. Cell transfection, cell supernatant medium passages and demonstration of viral replication were performed using BHK-21 cells as described above. From the first passage, a clear CPE was systematically observed in all experiments between days 4–7 post-inoculation except for chimeric PF/MART viruses and average amounts of viral RNA as well as infectious titres were comparable with those observed with wild-type viruses (Table 1).

In conclusion, using the ISA procedure we have generated two reverse genetics systems for low-passage isolates of ZIKV belonging to both the epidemic Asian lineage and to the African lineage. Both reverse genetics systems are available for the scientific community from the EVAg website under reference no. 001N-01891 and 001N-01890 for the PF and the DAK strains respectively (http://www.european-virus-archive.com/).

This work was initiated for the PF strain in December 2015. At that time, very few entire genome sequences of ZIKV with complete 5′ and 3′ noncoding regions were available in the Genbank database. Starting from these sequences, we designed and *de novo* synthesized three DNA fragments. Following many unsuccessful attempts to produce replicative virus, we sequenced the 5′ and 3′ noncoding regions of the PF ZIKV strain and identified 4 and 8 nt differences respectively within the 5′ and 3′ noncoding region sequences available in GenBank. Corrected DNA fragments were then designed and *de novo* synthesized, allowing the successful generation of infectious ZIKV. Subsequently, new sequences deposited in Genbank confirmed our findings. Since it was designed later, we did not encounter any of these problems with the reverse genetics for the DAK strain. This highlights the importance of the integrity of terminal non-coding flaviviral sequences for producing effective reverse genetics systems, as well as the benefit of a rapid sharing of genomic information during emergence situations.

IC technology is the most commonly used reverse genetics system. However, whilst ICs are powerful tools, construction and manipulation of DNA copies of full-length viral genomes in bacterial vectors can be laborious and time consuming, because they are often unstable and contain unwanted induced mutations^25^. We used in the present study the ISA method. Unlike other bacterium-free approaches, this method does not require any additional steps, such as cloning, propagation of cDNA into bacteria, or even RNA synthesis, other than the PCR amplification necessary to obtain overlapping DNA fragments. DNA fragments can be generated using a variety of initial sources including pre-existing infectious clones, viral RNA or *de novo* synthesized DNA genomic sequences. The use of pCMV enables viral RNA to be produced *in cellulo* after direct transfection and confers some technical advantages over the use of bacteriophage promoters that require the production of RNA *in vitro*. In addition, the ISA method also proved to be very suitable for the generation of genetically modified viruses^22^,^26^.

Here, we have developed two similar but independent reverse genetics systems for Asian and African wild-type ZIKV strains. The ISA technology allowed us to do so without any nucleotide modification of the genomic sequences. This is a great advantage for subsequent mutagenesis studies that will be operated in a completely preserved genomic backbone, allowing to safely identifying specific biological properties associated with genomic mutants.

Because both systems were designed following the same approach, they conveniently allowed producing chimeric ZIKV strains. With overlapping regions located exactly in the same genomic areas of PF and DAK viruses, the exchange of DNA fragments between both lineages was greatly facilitated. It revealed that a genomic backbone from one lineage of ZIKV, composed of 5′ and 3′ noncoding regions, structural genes and part of non-structural genes (NS4B and NS5) fully withstands the presence of non-structural genes from another ZIKV lineage. This information opens the way to further studies aiming at identifying genetic properties associated with genomic divergence observed in the different genes of both ZIKV lineages, as well as analyzing sequence constraints imposed on ZIKV genomes. Of note, we demonstrated that introducing a genetic fragment from any available ZIKV strains was possible using the ISA procedure, simply by amplifying the region of interest by RT-PCR from the viral genomic RNA.

In conclusion, we expect that the reverse genetics systems presented here will be directly applicable to and will greatly facilitate studies of pathophysiology or cell biology of ZIKV infection and genetic experiments aiming at therapeutics and vaccine development. Because they have been designed from low-passage ZIKV strains and a PF strain belonging to the outbreak lineage, the corresponding viruses can be used as appropriate models to study ZIKV infections both *in cellulo* or *in vivo*. In particular, these versatile systems should be ideal for evaluation of the direct effect of genetic changes observed in different evolutionary time-scales of ZIKV.

## Methods

### Cells

Human adrenal carcinoma SW13 cells were grown at 37 °C with 5% CO_2_ in RPMI Medium (Life technologies) supplemented with 10% heat-inactivated foetal bovine serum (FBS; Life Technologies) and 1% Penicillin/ Streptomycin (PS; 5000U mL^−1^ and 5000 μg mL^−1^; Life Technologies). Human embryonic kidney HEK-293 cells (ATCC number CCL-1573) were grown at 37 °C with 5% CO_2_ in a Minimal Essential Medium (MEM, Life Technologies) supplemented with 7% FBS, 1% PS and 1% Minimun Essential Medium Non-Essential Amino Acids (MEM NEAA; Life Technologies). Baby hamster kidney BHK-21 cells (ATCC number CCL-10) were grown at 37 °C with 5% CO_2_ in a MEM supplemented with 5% FBS, 5% TPB (Tryptose Phosphate Broth; Life Technologies), 1% L-Glutamine 200 mM (Life Technologies) and 1% PS. All cell culture experiments were performed in accordance with relevant guidelines and regulations.

### Viruses

ZIKV strain H/PF/2013, called ‘PF’ in the present study, was isolated in November 2013 from the serum of a 51-year-old female patient who was hospitalized in French Polynesia (available on http://www.european-virus-archive.com/ under reference no. 001v-EVA1545; GenBank accession number KJ776791)^27^. Before sequencing, PF ZIKV strain had been passaged three times in Vero cells. ZIKV strain A.taylori-tc/SEN/1984/41662-DAK, called ‘DAK’ in the present study, was isolated in December 1984 from *Aedes taylori* mosquito in Dakar, Senegal (GenBank accession number KU955592). Prior to deposition of the sequence of the DAK ZIKV strain it had been passaged once in AP61 *Aedes pseudoscutellaris* cells, once in C6/36 *Aedes albopictus* cells and three times in Vero cells. ZIKV strain MRS_OPY_Martinique_PaRi_2015, called ‘MART’ in the present study, was isolated in December 2015 from the serum of a 54-year-old woman who was hospitalized in Martinique (available on http://www.european-virus-archive.com/ under reference no. 001v-EVAg1589; GenBank accession number KU647676)^28^. Before sequencing, MART ZIKV strain had been passaged once in Vero cells. All EVAg products were provided in accordance with relevant guidelines and regulations.

### PCR amplification of overlapping DNA fragments

Primer and DNA fragment sequences are detailed in Supplementary Table S1 and Supplementary Note S1 respectively. Amplicons of PF and DAK ZIKV strains were produced using the Platinum PCR SuperMix High Fidelity kit (Life Technologies). The mixture (final volume, 50 μl) contained 45 μL of SuperMix, 2 μl of DNA template at 1 ng/μl (*De novo* synthesized DNA fragment; Synthetic genes were supplied in a standard pUC57 vector) and 1.5 μL of each primer (10 μM working solution were used). Assays were performed on a Biometra TProfessional Standard Gradient thermocycler with the following conditions: 94 °C for 2 min followed by 40 cycles of 94 °C for 15 s, 60 °C for 30 s, 68 °C for 5 min and a final elongation step of 68 °C for 10 min. For MART, the second cDNA fragment was generated by RT-PCR from clarified cell supernatant media. Viral RNA was extracted using the EZ1 Virus Mini kit v2.0 and the EZ1 advanced XL machine (both from Qiagen) according to the manufacturer’s instructions and amplified with the Superscript III One-Step RT-PCR Platinum Taq High Fidelity kit (Life Technologies). The mixture (final volume, 25 μl) contained 12.5 μl Reaction Mix, 2 μl nucleic acid extract, 1 μL of each primer (10 μM working solution were used), 1 μl Enzyme Mix. Assays were performed on a Biometra TProfessional Standard Gradient thermocycler with the following conditions: 50 °C for 30 min, 94 °C for 2 min followed by 40 cycles of 94 °C for 15 s, 64 °C for 30 s, 68 °C for 5 min. Size of the PCR products was verified by gel electrophoresis and purified using an Amicon Ultra 0.5 ml kit (Millipore).

### Cell transfection and cell supernatant passages

An equimolar mixture of the amplified DNA fragments was used for transfection. DNA-lipid complex was prepared as followed: 12 μl of Lipofectamine 3000 (Life Technologies) was diluted in 250 μL Opti-MEM medium (Life Technologies) and then mixed with a master solution of DNA which contained 3 μg of DNA and 6 μl of P3000 reagent diluted in 250 μL Opti-MEM medium. After an incubation period of 45 minutes at room temperature, the DNA-lipid complex was added to a 12.5 cm^2^ culture flask of subconfluent cells which contained 1 mL of medium without antibiotics. Each experiment was performed in quadruplicate. After an incubation period of 12 hours, the supernatant medium was removed, cells were washed twice (HBSS; Life Technologies) and 3 mL of fresh medium with antibiotic were added. Cells were then incubated for 7 days.

Cell supernatant media were then serially passaged twice using the same cell types. 333 μl of cell supernatant medium clarified by centrifugation was inoculated into a 12.5 cm^2^ culture flask of confluent cells. After an incubation period of 12 hours, cells were washed twice (HBSS) and 3 mL of fresh medium was added before incubation of 5–7 days.

### Real-time reverse transcriptase (RT) PCR assay

Quantification was performed using the GoTaq^®^ probe-1-step RT-qPCR system kit (Promega). A fragment of 187 nt (nucleotide position of PF ZIKV strain 2631 to 2809) was used to detect the genomic RNA of all ZIKVs. The mixture (final volume: 20 μl) contained 10 μl of Gotaq prob qPCR Master Mix, 0.5 μL of each primer (10 μM working solution were used; forward CTTGGAGTGCTTGTGATT; reverse CTCCTCCAGTGTTCATTT), 0.2 μl of probe (10 μM working solution was used; FAM-AAGAAGAGAATGACCACAAAGATCATC-TAMRA), 0.5 μl of Go script RT mix, 0.3 μl of nuclease-free water and 8 μl of viral RNA (extracted as described above). Assays were performed using the CFX96 Touch real-time PCR machine (Bio-Rad) with the following conditions: 50 °C for 15 min, 95 °C for 2 min, followed by 45 cycles of 95 °C for 15 s, 60 °C for 40 s. Data collection occurred during the 60 °C step. The amount of viral RNA was calculated from standard curves using synthetic RNA.

### Tissue Culture Infectious Dose 50 (TCID50) assay

We used a method previously described to perform TCID50 assays^29^. For each determination, a 96-well plate culture of confluent BHK-21 was used. Wells were inoculated with 150 μL of a mix containing 100 μL of medium and 50 μL of serial 10-fold dilutions of clarified cell supernatant media. Each row included 6 wells of the same dilution and two negative controls. Plates were incubated for 5 days and each well was read for absence or presence of CPE except for chimeric PF/MART viruses for which no CPE was observed at passage 2 in BHK-21 cells. For PF/MART viruses, we used a qRT-PCR assay (see above) as indicator of the presence of infectious virus: wells were considered as positive when amounts of viral RNA were higher than 10^6^ copies/mL while in all negative wells we detected amounts of viral RNA below 10^3^ copies/mL (amounts expected after the dilution of the initial RNA yields). The determination of the TCID50/ml was performed using the method of Reed and Muench^30^.

### Full-length genome sequencing

Verification of the complete genome integrity was performed at passage 2 (only one replicate per condition) as previously described^24^. Nucleic acid extracts from cell supernatants (extracted as described above) were used to amplify (Superscript III One-Step RT-PCR Platinum TaqHifi kit; Life Technologies) the complete viral genome in three fragments using a set of specific primers. Pooled amplicons were analyzed using the Ion PGM Sequencer (Life technologies) according to the manufacturer’s instructions. Using the CLC Genomics Workbench 6 software, read sequences were trimmed using quality score, by removing the primers used to amplify the complete genome by RT-PCR, by systematically removing 6 nt at the 5′ and 3′ termini and finally by discarding those with a length below 30 nt. Remaining reads were mapped using the expected sequence of the virus and mutation frequency at each position was calculated (number of reads with a mutation compared to the reference divided by the total number of reads; only mutations with frequency ≥5% were recorded).

### Virus replication kinetics

An moi of 0.01 was used to infect a 12.5 cm^2^ culture flask of confluent BHK-21 cells with recombinant or parental PF ZIKVs produced in BHK-21 cells. Cells were washed twice (HBSS) 3 hours after the infection and 4 mL of medium was added. 400 μL of cell supernatant media were sampled at 24, 48, 72 and 96 hours post-infection. They were clarified by centrifugation, aliquoted and stored at −80 °C. They were then analyzed using the real-time RT-PCR assay as described above. Each experiment was performed in triplicate.

## Additional Information

**How to cite this article**: Atieh, T. *et al*. Simple reverse genetics systems for Asian and African Zika viruses. *Sci. Rep.* **6**, 39384; doi: 10.1038/srep39384 (2016).

**Publisher's note:** Springer Nature remains neutral with regard to jurisdictional claims in published maps and institutional affiliations.

## Supplementary Material

Supplementary Information

## Figures and Tables

**Table 1:** Characteristics of the recovered ZIKVs.

Viral strain	Template for subgenomic amplicons*			Cell line used	CPE	Amounts of viral RNA (Log_10_ copies/mL; mean +/− SD)	Infectious titres (Log_10_ TCID50/mL; mean +/− SD)	Complete genome similarity
	I	II	III					
PF	PF-I	PF-II	PF-III	BHK-21	Yes	7.23 +/− 0.38	4.14 +/− 0.11	99.96%
				HEK-293	Yes	7.92 +/− 0.40	3.94 +/− 0.18	99.97%
				SW13	Yes	6.42 +/− 1.17	4.02 +/− 0.28	99.97%
DAK	DAK-I	DAK-II	DAK-III	BHK-21	Yes	6.74 +/− 0.37	5.19 +/− 0.18	99.96%
				HEK-293	Yes	7.43 +/− 0.20	5.19 +/− 0.18	99.98%
				SW13	No	6.82 +/− 0.15	5.02 +/− 0.28	99.95%
Chimeric PF/DAK	PF-I	DAK-I	PF-III	BHK-21	Yes	7.28 +/− 0.15	3.94 +/− 0.18	99.96%
Chimeric DAK/PF	DAK-I	PF-II	DAK-III	BHK-21	Yes	7.40 +/− 0.33	4.94 +/− 0.18	99.97%
Chimeric PF/MART	PF-I	MART-II	PF-III	BHK-21	No	7.03 +/− 0.64	5.27 +/− 0.07	99.96%

Template for production of the first (I), second (II) and third (III) overlapping DNA fragments, cell lines used for transfection and passages, quantification of the viral RNA and infectious titres in cell supernatant media at the second passage by real-time RT-PCR and TCID50 assay, respectively, presence or absence of cytopathic effect (CPE) and complete genome similarity are summarized. Viral RNA and infectious titer quantifications were expressed as mean +/− SD of log_10_ values (each experiment was performed in quadruplicate).

^*^All subgenomic amplicons were produced using *de novo* synthesized DNA fragment as templates except for MART-II which was generated by RT-PCR from the cell culture supernatant medium of the MART ZIKV strain.
