# Supplementary material for: Simple reverse genetics systems for Asian and African Zika viruses
Source: Sci Rep. 2016 Dec 19;6:39384. doi: 10.1038/srep39384 (PMC5171905; doi:10.1038/srep39384)
Supplement: Supplementary Information [file srep39384-s1.pdf]

Supplementary Information

Simple reverse genetics systems for Asian and African Zika viruses

Thérèse Atieh[1], Cécile Baronti[1], Xavier de Lamballerie[1,2] and Antoine Nougairède[1,2,*]

[1] UMR "Emergence des Pathologies Virales" (EPV: Aix-Marseille university - IRD 190 - Inserm 1207 - EHESP), Marseille, 13385, France

[2] Institut hospitalo-universitaire Méditerranée-Infection, Marseille, 13385, France

Supplementary Table S1: Primers used for the amplification of the different DNA fragments

Strain	DNA fragment	Primer Forward	Position*	Primer Reverse	Position*
PF/MART	I	CACCCAACTGATCTTCAGCATCT	¶	GCAGCACCATTCCTCGATCACC	3407-3428
	II	GAAACATGTGGAACAAGAGGACCA	3354-3377	GAAGCTCCAGCCAAGTAACTTC	7600-7621
	III	GTACTGGAACTCCTCTACAGCCA	7553-7575	CTCAGGGTCAATGCCAGCGCTT	£
DAK	I	CACCCAACTGATCTTCAGCATCT	¶	GCAGCACCATTCCTCTATGACC	3407-3428
	II	CACGTGGAGGAGACATGCGGAAC	3345-3367	GTGTAAATAAGGGAAGCCCCTGC	7611-7633
	III	GTACTGGAACTCCTCCACAGCCA	7553-7575	CTCAGGGTCAATGCCAGCGCTT	£

Primers located respectively at the 5′ and 3′ terminus of the pCMV (¶) and the HDR/SV40pA (£).
* All positions based on complete PF strain sequence.

Supplementary Note S1: Nucleotide sequence of the DNA fragments used for the ISA procedure

PF-I
CACCCAACTGATCTTCAGCATCTTCAATATTGGCCATTAGCCATATTATTCATTGGTTATATAGCATAAATCAATAT
TGGCTATTGGCCATTGCATACGTTGTATCTATATCATAATATGTACATTTATATTGGCTCATGTCCAATATGACCGCC
ATGTTGGCATTGATTATTGACTAGTTATTAATAGTAATCAATTACGGGGTCATTAGTTCATAGCCCATATATGGAGT
TCCGCGTTACATAACTTACGGTAAATGGCCCGCCTGGCTGACCGCCCAACGACCCCCGCCCATTGACGTCAATAAT
GACGTATGTTCCCATAGTAACGCCAATAGGGACTTTCCATTGACGTCAATGGGTGGAGTATTTACGGTAAACTGCC
CACTTGGCAGTACATCAAGTGTATCATATGCCAAGTCCGCCCCCTATTGACGTCAATGACGGTAAATGGCCCGCCT
GGCATTATGCCCAGTACATGACCTTACGGGACTTTCCTACTTGGCAGTACATCTACGTATTAGTCATCGCTATTACC
ATGGTGATGCGGTTTTGGCAGTACACCAATGGGCGTGGATAGCGGTTTGACTCACGGGGATTTCCAAGTCTCCACC
CCATTGACGTCAATGGGAGTTTGTTTTGGCACCAAAATCAACGGGACTTTCCAAAATGTCGTAATAACCCCGCCCC
GTTGACGCAAATGGGCGGTAGGCGTGTACGGTGGGAGGTCTATATAAGCAGAGCTCGTTTAGTGAACCGAGTTGTT
GATCTGTGTGAATCAGACTGCGACAGTTCGAGTTTGAAGCGAAAGCTAGCAACAGTATCAACAGGTTTTATTTTGG
ATTTGGAAACGAGAGTTTCTGGTCATGAAAAACCCAAAAAAGAAATCCGGAGGATTCCGGATTGTCAATATGCTAA
AACGCGGAGTAGCCCGTGTGAGCCCCTTTGGGGGCTTGAAGAGGCTGCCAGCCGGACTTCTGCTGGGTCATGGGCC
CATCAGGATGGTCTTGGCGATTCTAGCCTTTTTGAGATTCACGGCAATCAAGCCATCACTGGGTCTCATCAATAGAT
GGGGTTCAGTGGGGAAAAAAGAGGCTATGGAAATAATAAAGAAGTTCAAGAAAGATCTGGCTGCCATGCTGAGAA
TAATCAATGCTAGGAAGGAGAAGAAGAGACGAGGCGCAGATACTAGTGTCGGAATTGTTGGCCTCCTGCTGACCA
CAGCTATGGCAGCGGAGGTCACTAGACGTGGGAGTGCATACTATATGTACTTGGACAGAAACGACGCTGGGGAGG
CCATATCTTTTCCAACCACATTGGGGATGAATAAGTGTTATATACAGATCATGGATCTTGGACACATGTGTGATGCC
ACCATGAGCTATGAATGCCCTATGCTGGATGAGGGGGTGGAACCAGATGACGTCGATTGTTGGTGCAACACGACGT
CAACTTGGGTTGTGTACGGAACCTGCCATCACAAAAAAGGTGAAGCACGGAGATCTAGAAGAGCTGTGACGCTCC
CCTCCCATTCCACTAGGAAGCTGCAAACGCGGTCGCAAACCTGGTTGGAATCAAGAGAATACACAAAGCACTTGAT
TAGAGTCGAAAATTGGATATTCAGGAACCCTGGCTTCGCGTTAGCAGCAGCTGCCATCGCTTGGCTTTTGGGAAGC
TCAACGAGCCAAAAAGTCATATACTTGGTCATGATACTGCTGATTGCCCCGGCATACAGCATCAGGTGCATAGGAG
TCAGCAATAGGGACTTTGTGGAAGGTATGTCAGGTGGGACTTGGGTTGATGTTGTCTTGGAACATGGAGGTTGTGT
CACCGTAATGGCACAGGACAAACCGACTGTCGACATAGAGCTGGTTACAACAACAGTCAGCAACATGGCGGAGGT
AAGATCCTACTGCTATGAGGCATCAATATCGGACATGGCTTCGGACAGCCGCTGCCCAACACAAGGTGAAGCCTAC
CTTGACAAGCAATCAGACACTCAATATGTCTGCAAAAGAACGTTAGTGGACAGAGGCTGGGGAAATGGATGTGGA
CTTTTTGGCAAAGGGAGCCTGGTGACATGCGCTAAGTTTGCATGCTCCAAGAAAATGACCGGGAAGAGCATCCAGC

CAGAGAATCTGGAGTACCGGATAATGCTGTCAGTTCATGGCTCCCAGCACAGTGGGATGATCGTTAATGACACAGG
ACATGAAACTGATGAGAATAGAGCGAAGGTTGAGATAACGCCCAATTCACCAAGAGCCGAAGCCACCCTGGGGGG
TTTTGGAAGCCTAGGACTTGATTGTGAACCGAGGACAGGCCTTGACTTTTCAGATTTGTATTACTTGACTATGAATA
ACAAGCACTGGTTGGTTCACAAGGAGTGGTTCCACGACATTCCATTACCTTGGCACGCTGGGGCAGACACCGGAAC
TCCACACTGGAACAACAAAGAAGCACTGGTAGAGTTCAAGGACGCACATGCCAAAAGGCAAACTGTCGTGGTTCT
AGGGAGTCAAGAAGGAGCAGTTCACACGGCCCTTGCTGGAGCTCTGGAGGCTGAGATGGATGGTGCAAAGGGAAG
GCTGTCCTCTGGCCACTTGAAATGTCGCCTGAAAATGGATAAACTTAGATTGAAGGGCGTGTCATACTCCTTGTGTA
CCGCAGCGTTCACATTCACCAAGATCCCGGCTGAAACACTGCACGGGACAGTCACAGTGGAGGTACAGTACGCAG
GGACAGATGGACCTTGCAAGGTTCCAGCTCAGATGGCGGTGGACATGCAAACTCTGACCCCAGTTGGGAGGTTGAT
AACCGCTAACCCCGTAATCACTGAAAGCACTGAGAACTCTAAGATGATGCTGGAACTTGATCCACCATTTGGGGAC
TCTTACATTGTCATAGGAGTCGGGGAGAAGAAGATCACCCACCACTGGCACAGGAGTGGCAGCACCATTGGAAAA
GCATTTGAAGCCACTGTGAGAGGTGCCAAGAGAATGGCAGTCTTGGGAGACACAGCCTGGGACTTTGGATCAGTTG
GAGGCGCTCTCAACTCATTGGGCAAGGGCATCCATCAAATTTTTGGAGCAGCTTTCAAATCATTGTTTGGAGGAAT
GTCCTGGTTCTCACAAATTCTCATTGGAACGTTGCTGATGTGGTTGGGTCTGAACACAAAGAATGGATCTATTTCCC
TTATGTGCTTGGCCTTAGGGGGAGTGTTGATCTTCTTATCCACAGCTGTCTCTGCTGATGTGGGGTGCTCGGTGGAC
TTCTCAAAGAAGGAGACGAGATGCGGTACAGGGGTGTTCGTCTATAACGACGTTGAAGCCTGGAGGGACAGGTAC
AAGTACCATCCTGACTCCCCCCGTAGATTGGCAGCAGCAGTCAAGCAAGCCTGGGAAGATGGTATCTGTGGGATCT
CCTCTGTTTCAAGAATGGAAAACATCATGTGGAGATCAGTAGAAGGGGAGCTCAACGCAATCCTGGAAGAGAATG
GAGTTCAACTGACGGTCGTTGTGGGATCTGTAAAAAACCCCATGTGGAGAGGTCCACAGAGATTGCCCGTGCCTGT
GAACGAGCTGCCCCACGGCTGGAAGGCTTGGGGGAAATCGTACTTCGTCAGAGCAGCAAAGACAAATAACAGCTT
TGTCGTGGATGGTGACACACTGAAGGAATGCCCACTCAAACATAGAGCATGGAACAGCTTTCTTGTGGAGGATCAT
GGGTTCGGGGTATTTCACACTAGTGTCTGGCTCAAGGTTAGAGAAGATTATTCATTAGAGTGTGATCCAGCCGTTAT
TGGAACAGCTGTTAAGGGAAAGGAGGCTGTACACAGTGATCTAGGCTACTGGATTGAGAGTGAGAAGAATGACAC
ATGGAGGCTGAAGAGGGCCCATCTGATCGAGATGAAAACATGTGAATGGCCAAAGTCCCACACATTGTGGACAGA
TGGAATAGAAGAGAGTGATCTGATCATACCCAAGTCTTTAGCTGGGCCACTCAGCCATCACAATACCAGAGAGGGC
TACAGGACCCAAATGAAAGGGCCATGGCACAGTGAAGAGCTTGAAATTCGGTTTGAGGAATGCCCAGGCACTAAG
GTCCACGTGGAGGAAACATGTGGAACAAGAGGACCATCTCTGAGATCAACCACTGCAAGCGGAAGGGTGATCGAG
GAATGGTGCTGC

PF-II

GAAACATGTGGAACAAGAGGACCATCTCTGAGATCAACCACTGCAAGCGGAAGGGTGATCGAGGAATGGTGCTGC
AGGGAGTGCACAATGCCCCCACTGTCGTTCCGGGCTAAAGATGGCTGTTGGTATGGAATGGAGATAAGGCCCAGG
AAAGAACCAGAAAGTAACTTAGTAAGGTCAATGGTGACTGCAGGATCAACTGATCACATGGATCACTTCTCCCTTG
GAGTGCTTGTGATTCTGCTCATGGTGCAGGAAGGGCTGAAGAAGAGAATGACCACAAAGATCATCATAAGCACAT
CGATGGCAGTGCTGGTAGCTATGATCCTGGGAGGATTTTCAATGAGTGACCTGGCTAAGCTTGCAATTTTGATGGG
TGCCACCTTCGCGGAAATGAACACTGGAGGAGATGTAGCTCATCTGGCGCTGATAGCGGCATTCAAAGTCAGACCA
GCGTTGCTGGTATCTTTCATCTTCAGAGCTAATTGGACACCCCGTGAAAGCATGCTGCTGGCCTTGGCCTCGTGTCT
TTTGCAAACTGCGATCTCCGCCTTGGAAGGCGACCTGATGGTTCTCATCAATGGTTTTGCTTTGGCCTGGTTGGCAA
TACGAGCGATGGTTGTTCCACGCACTGATAACATCACCTTGGCAATCCTGGCTGCTCTGACACCACTGGCCCGGGG
CACACTGCTTGTGGCGTGGAGAGCAGGCCTTGCTACTTGCGGGGGGTTTATGCTCCTCTCTCTGAAGGGAAAAGGC
AGTGTGAAGAAGAACTTACCATTTGTCATGGCCCTGGGACTAACCGCTGTGAGGCTGGTCGACCCCATCAACGTGG
TGGGACTGCTGTTGCTCACAAGGAGTGGGAAGCGGAGCTGGCCCCCTAGCGAAGTACTCACAGCTGTTGGCCTGAT
ATGCGCATTGGCTGGAGGGTTCGCCAAGGCAGATATAGAGATGGCTGGGCCCATGGCCGCGGTCGGTCTGCTAATT
GTCAGTTACGTGGTCTCAGGAAAGAGTGTGGACATGTACATTGAAAGAGCAGGTGACATCACATGGGAAAAAGAT
GCGGAAGTCACTGGAAACAGTCCCCGGCTCGATGTGGCGCTAGATGAGAGTGGTGATTTCTCCCTGGTGGAGGATG
ACGGTCCCCCCATGAGAGAGATCATACTCAAGGTGGTCCTGATGACCATCTGTGGCATGAACCCAATAGCCATACC
CTTTGCAGCTGGAGCGTGGTACGTATACGTGAAGACTGGAAAAAGGAGTGGTGCTCTATGGGATGTGCCTGCTCCC
AAGGAAGTAAAAAAGGGGGAGACCACAGATGGAGTGTACAGAGTAATGACTCGTAGACTGCTAGGTTCAACACAA
GTTGGAGTGGGAGTTATGCAAGAGGGGGTCTTTCACACTATGTGGCACGTCACAAAAGGATCCGCGCTGAGAAGC
GGTGAAGGGAGACTTGATCCATACTGGGGAGATGTCAAGCAGGATCTGGTGTCATACTGTGGTCCATGGAAGCTAG
ATGCCGCCTGGGACGGGCACAGCGAGGTGCAGCTCTTGGCCGTGCCCCCCGGAGAGAGAGCGAGGAACATCCAGA
CTCTGCCCGGAATATTTAAGACAAAGGATGGGGACATTGGAGCGGTTGCGCTGGATTACCCAGCAGGAACTTCAGG
ATCTCCAATCCTAGACAAGTGTGGGAGAGTGATAGGACTTTATGGCAATGGGGTCGTGATCAAAAATGGGAGTTAT
GTTAGTGCCATCACCCAAGGGAGGAGGGAGGAAGAGACTCCTGTTGAGTGCTTCGAGCCTTCGATGCTGAAGAAG
AAGCAGCTAACTGTCTTAGACTTGCATCCTGGAGCTGGGAAAACCAGGAGAGTTCTTCCTGAAATAGTCCGTGAAG
CCATAAAAACAAGACTCCGTACTGTGATCTTAGCTCCAACCAGGGTTGTCGCTGCTGAAATGGAGGAAGCCCTTAG
AGGGCTTCCAGTGCGTTATATGACAACAGCAGTCAATGTCACCCACTCTGGAACAGAAATCGTCGACTTAATGTGC

CATGCCACCTTCACTTCACGTCTACTACAGCCAATCAGAGTCCCCAACTATAATCTGTATATTATGGATGAGGCCCA
CTTCACAGATCCCTCAAGTATAGCAGCAAGAGGATACATTTCAACAAGGGTTGAGATGGGCGAGGCGGCTGCCATC
TTCATGACCGCCACGCCACCAGGAACCCGTGACGCATTTCCGGACTCCAACTCACCAATTATGGACACCGAAGTGG
AAGTCCCAGAGAGAGCCTGGAGCTCAGGCTTTGATTGGGTGACGGATCATTCTGGAAAAACAGTTTGGTTTGTTCC
AAGCGTGAGGAACGGCAATGAGATCGCAGCTTGTCTGACAAAGGCTGGAAAACGGGTCATACAGCTCAGCAGAAA
GACTTTTGAGACAGAGTTCCAGAAAACAAAACATCAAGAGTGGGACTTTGTCGTGACAACTGACATTTCAGAGATG
GGCGCCAACTTTAAAGCTGACCGTGTCATAGATTCCAGGAGATGCCTAAAGCCGGTCATACTTGATGGCGAGAGAG
TCATTCTGGCTGGACCCATGCCTGTCACACATGCCAGCGCTGCCCAGAGGAGGGGGCGCATAGGCAGGAATCCCAA
CAAACCTGGAGATGAGTATCTGTATGGAGGTGGGTGCGCAGAGACTGACGAAGACCATGCACACTGGCTTGAAGC
AAGAATGCTCCTTGACAATATTTACCTCCAAGATGGCCTCATAGCCTCGCTCTATCGACCTGAGGCCGACAAAGTA
GCAGCCATTGAGGGAGAGTTCAAGCTTAGGACGGAGCAAAGGAAGACCTTTGTGGAACTCATGAAAAGAGGAGAT
CTTCCTGTTTGGCTGGCCTATCAGGTTGCATCTGCCGGAATAACCTACACAGATAGAAGATGGTGCTTTGATGGCAC
GACCAACAACACCATAATGGAAGACAGTGTGCCGGCAGAGGTGTGGACCAGACACGGAGAGAAAAGAGTGCTCA
AACCGAGGTGGATGGACGCCAGAGTTTGTTCAGATCATGCGGCCCTGAAGTCATTCAAGGAGTTTGCCGCTGGGAA
AAGAGGAGCGGCTTTTGGAGTGATGGAAGCCCTGGGAACACTGCCAGGACACATGACAGAGAGATTCCAGGAAGC
CATTGACAACCTCGCTGTGCTCATGCGGGCAGAGACTGGAAGCAGGCCTTACAAAGCCGCGGCGGCCCAATTGCCG
GAGACCCTAGAGACCATTATGCTTTTGGGGTTGCTGGGAACAGTCTCGCTGGGAATCTTTTTCGTCTTGATGAGGAA
CAAGGGCATAGGGAAGATGGGCTTTGGAATGGTGACTCTTGGGGCCAGCGCATGGCTCATGTGGCTCTCGGAAATT
GAGCCAGCCAGAATTGCATGTGTCCTCATTGTTGTGTTCCTATTGCTGGTGGTGCTCATACCTGAGCCAGAAAAGCA
AAGATCTCCCCAGGACAACCAAATGGCAATCATCATCATGGTAGCAGTAGGTCTTCTGGGCTTGATTACCGCCAAT
GAACTCGGATGGTTGGAGAGAACAAAGAGTGACCTAAGCCATCTAATGGGAAGGAGAGAGGAGGGGGCAACCAT
AGGATTCTCAATGGACATTGACCTGCGGCCAGCCTCAGCTTGGGCCATCTATGCTGCCTTGACAACTTTCATTACCC
CAGCCGTCCAACATGCAGTGACCACTTCATACAACAACTACTCCTTAATGGCGATGGCCACGCAAGCTGGAGTGTT
GTTTGGTATGGGCAAAGGGATGCCATTCTACGCATGGGACTTTGGAGTCCCGCTGCTAATGATAGGTTGCTACTCA
CAATTAACACCCCTGACCCTAATAGTGGCCATCATTTTGCTCGTGGCGCACTACATGTACTTGATCCCAGGGCTGCA
GGCAGCAGCTGCGCGTGCTGCCCAGAAGAGAACGGCAGCTGGCATCATGAAGAACCCTGTTGTGGATGGAATAGT
GGTGACTGACATTGACACAATGACAATTGACCCCCAAGTGGAGAAAAAGATGGGACAGGTGCTACTCATAGCAGT
AGCCGTCTCCAGCGCCATACTGTCGCGGACCGCCTGGGGGTGGGGGGAGGCTGGGGCCCTGATCACAGCGGCAAC
TTCCACTTTGTGGGAAGGCTCTCCGAACAAGTACTGGAACTCCTCTACAGCCACTTCACTGTGTAACATTTTTAGGG
GAAGTTACTTGGCTGGAGCTTC

PF-III

GTACTGGAACTCCTCTACAGCCACTTCACTGTGTAACATTTTTAGGGGAAGTTACTTGGCTGGAGCTTCTCTAATCT
ACACAGTAACAAGAAACGCTGGCTTGGTCAAGAGACGTGGGGGTGGAACAGGAGAGACCCTGGGAGAGAAATGG
AAGGCCCGCTTGAACCAGATGTCGGCCCTGGAGTTCTACTCCTACAAAAAGTCAGGCATCACCGAGGTGTGCAGAG
AAGAGGCCCGCCGCGCCCTCAAGGACGGTGTGGCAACGGGAGGCCATGCTGTGTCCCGAGGAAGTGCAAAGCTGA
GATGGTTGGTGGAGCGGGGATACCTGCAGCCCTATGGAAAGGTCATTGATCTTGGATGTGGCAGAGGGGGCTGGA
GTTACTACGCCGCCACCATCCGCAAAGTTCAAGAAGTGAAAGGATACACAAAAGGAGGCCCTGGTCATGAAGAAC
CCATGTTGGTGCAAAGCTATGGGTGGAACATAGTCCGTCTTAAGAGTGGGGTGGACGTCTTTCATATGGCGGCTGA
GCCGTGTGACACGTTGCTGTGTGACATAGGTGAGTCATCATCTAGTCCTGAAGTGGAAGAAGCACGGACGCTCAGA
GTCCTCTCCATGGTGGGGGATTGGCTTGAAAAAAGACCAGGAGCCTTTTGTATAAAAGTGTTGTGCCCATACACCA
GCACTATGATGGAAACCCTGGAGCGACTGCAGCGTAGGTATGGGGGAGGACTGGTCAGAGTGCCACTCTCCCGCA
ACTCTACACATGAGATGTACTGGGTCTCTGGAGCGAAAAGCAACACCATAAAAAGTGTGTCCACCACGAGCCAGCT
CCTCTTGGGGCGCATGGACGGGCCCAGGAGGCCAGTGAAATATGAGGAGGATGTGAATCTCGGCTCTGGCACGCG
GGCTGTGGTAAGCTGCGCTGAAGCTCCCAACATGAAGATCATTGGTAACCGCATTGAAAGGATCCGCAGTGAGCAC
GCGGAAACGTGGTTCTTTGACGAGAACCACCCATATAGGACATGGGCTTACCATGGAAGCTATGAGGCCCCCACAC
AAGGGTCAGCGTCCTCTCTAATAAACGGGGTTGTCAGGCTCCTGTCAAAACCCTGGGATGTGGTGACTGGAGTCAC
AGGAATAGCCATGACCGACACCACACCGTATGGTCAGCAAAGAGTTTTCAAGGAAAAAGTGGACACTAGGGTGCC
AGACCCCCAAGAAGGCACTCGTCAGGTTATGAGCATGGTCTCTTCCTGGTTGTGGAAAGAGCTAGGCAAACACAAA
CGGCCACGAGTCTGTACCAAAGAAGAGTTCATCAACAAGGTTCGTAGCAATGCAGCATTAGGGGCAATATTTGAA
GAGGAAAAAGAGTGGAAGACTGCAGTGGAAGCTGTGAACGATCCAAGGTTCTGGGCTCTAGTGGACAAGGAAAG
AGAGCACCACCTGAGAGGAGAGTGCCAGAGTTGTGTGTACAACATGATGGGAAAAAGAGAAAAGAAACAAGGGG
AATTTGGAAAGGCCAAGGGCAGCCGCGCCATCTGGTATATGTGGCTAGGGGCTAGATTTCTAGAGTTCGAAGCCCT
TGGATTCTTGAACGAGGATCACTGGATGGGGAGAGAGAACTCAGGAGGTGGTGTTGAAGGGCTGGGATTACAAAG
ACTCGGATATGTCCTAGAAGAGATGAGTCGCATACCAGGAGGAAGGATGTATGCAGATGACACTGCTGGCTGGGA
CACCCGCATCAGCAGGTTTGATCTGGAGAATGAAGCTCTAATCACCAACCAAATGGAGAAAGGGCACAGGGCCTT
GGCATTGGCCATAATCAAGTACACATACCAAAACAAAGTGGTAAAGGTCCTTAGACCAGCTGAAAAAGGGAAGAC

AGTTATGGACATTATTTCGAGACAAGACCAAAGGGGGAGCGGACAAGTTGTCACTTACGCTCTTAACACATTTACC
AACCTAGTGGTGCAACTCATTCGGAATATGGAGGCTGAGGAAGTTCTAGAGATGCAAGACTTGTGGCTGCTGCGGA
GGTCAGAGAAAGTGACCAACTGGTTGCAGAGCAACGGATGGGATAGGCTCAAACGAATGGCAGTCAGTGGAGATG
ATTGCGTTGTGAAGCCAATTGATGATAGGTTTGCACATGCCCTCAGGTTCTTGAATGATATGGGAAAAGTTAGGAA
GGACACACAAGAGTGGAAACCCTCAACTGGATGGGACAACTGGGAAGAAGTTCCGTTTTGCTCCCACCACTTCAAC
AAGCTCCATCTCAAGGACGGGAGGTCCATTGTGGTTCCCTGCCGCCACCAAGATGAACTGATTGGCCGGGCCCGCG
TCTCTCCAGGGGCGGGATGGAGCATCCGGGAGACTGCTTGCCTAGCAAAATCATATGCGCAAATGTGGCAGCTCCT
TTATTTCCACAGAAGGGACCTCCGACTGATGGCCAATGCCATTTGTTCATCTGTGCCAGTTGACTGGGTTCCAACTG
GGAGAACTACCTGGTCAATCCATGGAAAGGGAGAATGGATGACCACTGAAGACATGCTTGTGGTGTGGAACAGAG
TGTGGATTGAGGAGAACGACCACATGGAAGACAAGACCCCAGTTACGAAATGGACAGACATTCCCTATTTGGGAA
AAAGGGAAGACTTGTGGTGTGGATCTCTCATAGGGCACAGACCGCGCACCACCTGGGCTGAGAACATTAAAAACA
CAGTCAACATGGTGCGCAGGATCATAGGTGATGAAGAAAAGTACATGGACTACCTATCCACCCAAGTTCGCTACTT
GGGTGAAGAAGGGTCTACACCTGGAGTGCTGTAAGCACCAATCTTAGTGTTGTCAGGCCTGCTAGTCAGCCACAGC
TTGGGGAAAGCTGTGCAGCCTGTGACCCCCCCAGGAGAAGCTGGGAAACCAAGCCTATAGTCAGGCCGAGAACGC
CATGGCACGGAAGAAGCCATGCTGCCTGTGAGCCCCTCAGAGGACACTGAGTCAAAAAACCCCACGCGCTTGGAG
GCGCAGGATGGGAAAAGAAGGTGGCGACCTTCCCCACCCTTCAATCTGGGGCCTGAACTGGAGATCAGCTGTGGA
TCTCCAGAAGAGGGACTAGTGGTTAGAGGAGACCCCCCGGAAAACGCAAAACAGCATATTGACGCTGGGAAAGAC
CAGAGACTCCATGAGTTTCCACCACGCTGGCCGCCAGGCACAGATCGCCGAATAGCGGCGGCCGGTGTGGGGAAA
TCCATGGGTCTGGCCGGCATGGTCCCAGCCTCCTCGCTGGCGCCGGCTGGGCAACATTCCGAGGGGACCGTCCCCT
CGGTAATGGCGAATGGGACTCGCGACAGACATGATAAGATACATTGATGAGTTTGGACAAACCACAACTAGAATG
CAGTGAAAAAAATGCTTTATTTGTGAAATTAAGCGCTGGCATTGACCCTGAG

DAK-I

CACCCAACTGATCTTCAGCATCTTCAATATTGGCCATTAGCCATATTATTCATTGGTTATATAGCATAAATCAATAT
TGGCTATTGGCCATTGCATACGTTGTATCTATATCATAATATGTACATTTATATTGGCTCATGTCCAATATGACCGCC
ATGTTGGCATTGATTATTGACTAGTTATTAATAGTAATCAATTACGGGGTCATTAGTTCATAGCCCATATATGGAGT
TCCGCGTTACATAACTTACGGTAAATGGCCCGCCTGGCTGACCGCCCAACGACCCCCGCCCATTGACGTCAATAAT
GACGTATGTTCCCATAGTAACGCCAATAGGGACTTTCCATTGACGTCAATGGGTGGAGTATTTACGGTAAACTGCC
CACTTGGCAGTACATCAAGTGTATCATATGCCAAGTCCGCCCCCTATTGACGTCAATGACGGTAAATGGCCCGCCT
GGCATTATGCCCAGTACATGACCTTACGGGACTTTCCTACTTGGCAGTACATCTACGTATTAGTCATCGCTATTACC
ATGGTGATGCGGTTTTGGCAGTACACCAATGGGCGTGGATAGCGGTTTGACTCACGGGGATTTCCAAGTCTCCACC
CCATTGACGTCAATGGGAGTTTGTTTTGGCACCAAAATCAACGGGACTTTCCAAAATGTCGTAATAACCCCGCCCC
GTTGACGCAAATGGGCGGTAGGCGTGTACGGTGGGAGGTCTATATAAGCAGAGCTCGTTTAGTGAACCGAGTTGTT
GATCTGTGTGAATCAGACTGCGACAGTTCGAGTTTGAAGCGAGAGCTAACAACAGTATCAACAGGTTTAATTTGGA
TTTGGAAACGAGAGTTTCTGGTCATGAAAAACCCAAAGAAGAAATCCGGAGGATTCCGGATTGTCAATATGCTAAA
ACGCGGAGTAGCCCGTGTAAACCCCTTGGGGGGTTTGAAGAGGCTGCCAGCCGGACTTCTGCTGGGCCATGGACCC
ATCAGAATGGTTTTGGCGATACTAGCCTTCTTGAGATTCACAGCAATCAAACCATCACTGGGCCTCATCAATAGAT
GGGGTTCCGTGGGGAAGAAGGAGGCTATGGAAATAATAAAAAAGTTCAAGAAAGACCTTGCTGCCATGTTGAGAA
TTATCAATGCTAGGAAGGAGAGGAAGAGACGTGGCGCTGACACCAGCATCGGAATCGTCGGCCTCCTGCTGACCA
CAGCCATGGCAGCCGAGATCACTAGACGTGGGAGTGCATACTACATGTACTTGGACAGGAGCGATGCTGGTAAGG
CCATTTCTTTTGCCACCACATTGGGGGTGAACAAATGCCATGTACAGATCATGGACCTCGGGCACATGTGTGACGC
CACCATGAGTTATGAGTGCCCCATGCTAGACGAGGGAGTGGAGCCAGATGACGTCGATTGCTGGTGCAACACGAC
ATCGACTTGGGTTGTGTACGGAACCTGTCATCATAAAAAAGGTGAAGCACGACGATCCAGAAGAGCCGTGACGCTT
CCTTCTCACTCCACGAGGAAGCTGCAAACGCGATCGCAGACTTGGCTAGAGTCAAGAGAATACACAAAGCACCTG
ATCAAGGTTGAAAATTGGATATTCAGGAACCCCGGGTTTGCGCTAGTGGCTGTAGCTATAGCCTGGCTCCTGGGAA
GCTCGACGAGCCAAAAAGTTATATACTTGGTCATGATATTGTTGATTGCCCCGGCATACAGCATTAGGTGCATAGG
AGTTAGCAATAGAGACTTCGTGGAGGGCATGTCAGGTGGGACCTGGGTTGATGTTGTCTTGGAACATGGGGGTTGC
GTCACCGTGATGGCACAGGACAAGCCAACAGTTGATATCGAGTTGGTCACGACAACGGTTAGCAACATGGCCGAG
GTAAGATCCTACTGCTATGAGGCATCAATATCGGACATGGCTTCGGACAGCCGTTGTCCAACACAAGGTGAAGCCT
ACCTTGACAAGCAGTCAGACACTCAATATGTCTGCAAGAGAACATTGGTGGATAGAGGTTGGGGAAATGGGTGTG
GACTTTTTGGCAAAGGGAGCTTGGTGACATGTGCCAAGTTTACGTGCTCCAAGAAAATGACAGGCAAGAGCATCCA
GCCGGAGAACTTGGAGTACCGGATAATGCTATCAGTGCATGGATCCCAGCACAGTGGGATGATTGTGAATGACAC
AGGACATGAAACTGACGAAAACAGAGCAAAAGTCGAGGTCACACCCAATTCACCAAGAGCAGAAGCAACCTTGG
GAGGTTTTGGAAGCTTGGGACTTGACTGTGAACCAAGGACAGGCCTTGACTTCTCAGATCTGTATTACCTGACCAT
GAACAATAAGCATTGGTTGGTGCACAAGGAGTGGTTTCATGACATCCCATTACCTTGGCATGCTGGTGCAGACACT
GGAACTCCACACTGGAACAACAAAGAGGCATTGGTGGAGTTCAAGGACGCCCACGCCAAGAGGCAAACTGTTGTG
GTTCTGGGGAGCCAAGAGGGAGCTGTTCATACGGCCCTCGCTGGAGCTTTGGAGGCTGAGATGGATGGTGCAAAG
GGAAGGCTATTCTCTGGCCATTTGAAATGCCGCCTAAAAATGGACAAGCTTAGGTTGAAGGGTGTGTCATATTCCC

TGTGTACCGCAGCGTTCACATTTACCAAGGTCCCAGCTGAAACATTGCATGGAACAGTCACAGTGGAGGTGCAGTA
TGCAGGGACAGACGGACCCTGCAAAGTCCCAGCCCAGATGGCGGTGGACATGCAGACCCTGACCCCAGTTGGAAG
GCTGATAACCGCCAATCCTGTGATCACTGAAAGTACTGAGAATTCAAAGATGATGTTGGAGCTCGACCCACCATTT
GGGGATTCTTACATTGTCATAGGAGTCGGGGACAAGAAAATCACCCATCACTGGCATCGGAGTGGTAGCACCATCG
GAAAGGCATTTGAAGCCACTGTGAGAGGTGCCAAGAGAATGGCAGTCTTGGGGGACACAGCCTGGGACTTTGGAT
CAGTTGGGGGTGTGTTTAATTCATTGGGTAAGGGTATTCACCAGATCTTTGGAGCAGCTTTCAAATCACTGTTTGGA
GGAATGTCCTGGTTCTCACAGATCCTCATAGGCACACTGTTGGTGTGGTTAGGTTTGAACACAAAGAATGGATCTA
TCTCCCTCACATGCTTAGCCCTGGGGGGAGTGATGATCTTCCTTTCCACGGCTGTTTCTGCTGATGTTGGGTGCTCG
GTGGACTTCTCAAAAAAGGAAACGAGATGTGGCACGGGGGTGTTCGTCTACAATGACGTTGAAGCCTGGAGGGAC
CGGTACAAGTACCATCCTGACTCCCCCCGCAGATTGGCAGCAGCTGTTAAGCAGGCTTGGGAAGAGGGGATTTGTG
GGATCTCCTCCGTTTCGAGAATGGAAAACATCATGTGGAAATCAGTGGAAGGGGAGCTTAATGCAATCCTAGAGG
AGAATGGAGTTCAACTGACAGTTGTAGTGGGGTCTGTAAAAAACCCTATGTGGAGAGGTCCACAGAGATTGCCAGT
GCCTGTGAATGAGCTGCCCCATGGCTGGAAAGCCTGGGGGAAATCGTACTTTGTCAGAGCAGCAAAGACCAACAA
CAGTTTTGTTGTCGACGGTGACACACTGAAGGAGTGTCCGCTCAAACATAGAGCATGGAATAGCTTCCTTGTGGAG
GATCACGGGTTTGGGATCTTCCACACCAGTGTTTGGCTGAAGGTCAGAGAGGACTACTCACTAGAGTGTGACCCAG
CCGTCATAGGAACAGCTGTCAAGGGAAAGGAAGCTGCACACAGTGATCTAGGCTATTGGATTGAGAGTGAAAAGA
ATGACACATGGAGGCTGAGGAGGGCTCATCTGATTGAGATGAAAACATGTGAGTGGCCAAAGTCTCACACACTGT
GGACAGATGGAGTGGAAGAAAGTGATCTGATCATACCCAAGTCCTTAGCTGGTCCACTCAGCCACCACAACACCA
GAGAGGGTTACAGAACTCAAGTGAAAGGGCCATGGCATAGTGAAGAGCTCGAAATCCGGTTTGAGGAGTGCCCAG
GAACCAAGGTTCACGTGGAGGAGACATGCGGAACTAGAGGACCATCTCTGAGATCAACTACTGCTAGTGGAAGGG
TCATAGAGGAATGGTGCTGC

DAK-II

CACGTGGAGGAGACATGCGGAACTAGAGGACCATCTCTGAGATCAACTACTGCTAGTGGAAGGGTCATAGAGGAA
TGGTGCTGcAGGGAATGCACAATGCCTCCACTATCGTTCCGGGCGAAAGACGGCTGCTGGTATGGAATGGAGATAA
GGCCCAGAAAGGAACCAGAGAGCAACTTAGTGAGGTCCATGGTGACAGCAGGATCAACCGATCATATGGATCACT
TCTCTCTTGGAGTGCTTGTGATTCTACTCATGGTGCAGGAAGGTTTGAAGAAGAGAATGACCACAAAGATCATAAT
GAGCACATCAATGGCAGTGCTGGTAGCCATGGTCTTGGGAGGATTCTCAATGAGTGACCTGGCTAAGCTTGTGATC
CTGATGGGTGCTACTTTCGCAGAAATGAACACTGGAGGAGATGTAGCTCACTTGGCATTGGTTGCGGCATTTAAAG
TCAGACCAGCCTTGTTGGTTTCCTTCATCTTCAGAGCCAACTGGACACCCCGTGAGAGCATGCTGCTAGCCCTGGCT
TCGTGCCTCCTGCAGACTGCGATCTCTGCTCTTGAAGGCGAGCTGATGGTCCTCGTTAATGGATTTGCTTTGGCCTG
GTTGGCAATACGAGCAATGGCCGTGCCACGCACTGACAATATCGCTCTGGCAATTCTGGCTGCTCTAACACCATTA
GCCAGAGGCACACTGCTCGTGGCATGGAGAGCGGGCCTCGCCACTTGTGGAGGGTTCATGCTCCTTTCCCTGAAAG
GGAAAGGTAGTGTGAAGAAGAACCTGCCTTTTGTCATGGCCTTGGGGTTGACCGCTGTGAGGATAGTGGACCCCAT
CAATGTGGTAGGACTACTGTTACTCACAAGGAGTGGAAAACGGAGCTGGCCTCCTAGTGAAGTGCTTACAGCTGTT
GGCCTGATATGTGCACTGGCCGGAGGGTTTGCCAAGGCAGACATAGAGATGGCTGGGCCCATGGCTGCAGTGGGC
CTGCTAATTGTCAGTTATGTGGTCTCGGGAAAGAGTGTGGACATGTACATCGAAAGAGCAGGTGATATCACATGGG
AAAAAGACGCGGAAGTCACTGGAAACAGTCCTCGGCTTGACGTGGCACTAGATGAGAGTGGTGATTTCTCCTTGGT
AGAGGAGGATGGCCCACCCATGAGAGAGATCATACTCAAGGTGGTTCTGATGGCCATCTGTGGCATGAACCCAAT
AGCCATACCCTTCGCTGCAGGAGCGTGGTATGTGTATGTAAAGACTGGAAAAAGGAGTGGTGCCCTCTGGGACGTG
CCTGCTCCCAAAGAAGTGAAAAAAGGAGAGACTACAGATGGAGTGTACAGAGTGATGACTCGCAGACTGCTGGGT
TCAACACAGGTTGGAGTGGGAGTCATGCAAGAGGGAGTCTTCCACACCATGTGGCACGTCACAAAAGGAGCCGCA
TTGAGGAGCGGTGAAGGAAGACTTGATCCATATTGGGGGGACGTCAAGCAGGACTTGGTGTCATATTGTGGGCCTT
GGAAGTTGGATGCAGCCTGGGATGGACTAAGTGAAGTGCAGCTTTTGGCCGTACCCCCCGGAGAGAGGGCTAGAA
ACATTCAGACTCTGCCTGGAATATTCAAGACAAAGGATGGGGACATCGGAGCAGTCGCTCTAGACTACCCCGCAGG
AACCTCAGGATCTCCAATCCTAGACAAATGCGGAAGAGTGATAGGACTTTATGGCAATGGGGTTGTGATCAAGAAT
GGAAGCTATGTTAGTGCCATAACCCAGGGAAAAAGGGAGGAGGAGGCTCCAGTTGAGTGCTTTGAACCCTCGATG
CTGAGGAAGAAGCAGCTAACAGTCTTGGATCTGCATCCAGGAGCCGGGAAAACCAGGAGGGTTCTTCCTGAAATA
GTCCGTGAAGCCATAAAGAAGAGACTTCGCACAGTGATCCTAGCACCAACCAGGGTCGTTGCTGCTGAGATGGAG
GAAGCCCTAAGAGGACTGCCGGTGCGTTACATGACAACAGCAGTCAACGTCACCCACTCTGGGACAGAAATCGTC
GATTTGATGTGCCATGCCACCTTCACTTCACGCCTACTACAACCCATCAGAGTCCCCAACTACAACCTTTACATCAT
GGATGAAGCTCATTTCACAGATCCCTCAAGCATAGCTGCACGAGGATATATATCAACAAGGGTTGAAATGGGCGA
GGCGGCTGCTATCTTCATGACTGCTACACCACCAGGAACCCGCGATGCGTTTCCGGATTCCAACTCACCAATCATG
GACACAGAAGTGGAAGTTCCAGAGAGAGCCTGGAGCTCAGGCTTTGACTGGGTGACGGATCATTCTGGGAAAACA
ATTTGGTTTGTTCCAAGTGTGAGAAACGGAAATGAAATCGCAGCCTGTCTGACAAAGGCTGGAAAGCGGGTCATAC
AGCTCAGCAGGAAGACTTTTGAGACAGAGTTTCAGAAGACAAAAAATCAAGAGTGGGACTTTGTCATAACAACTG
ACATTTCAGAGATGGGTGCCAATTTCAAGGCTGACCGGGTCATAGATTCCAGGAGATGCCTAAAGCCAGTCATACT

CGATGGCGAGAGAGTCATCCTGGCTGGGCCCATGCCTGTCACGCATGCCAGTGCTGCTCAGAGGAGAGGACGTATA
GGCAGGAACCCCAACAAACCTGGAGATGAGTACATGTATGGAGGTGGGTGTGCAGAGACTGATGAAGACCATGCA
CACTGGCTTGAAGCAAGAATGCTTCTTGACAACATTTACCTCCAGGATGGCCTCATAGCCTCGCTCTATCGGCCTGA
GGCCGACAAGGTAGCCGCCATTGAGGGAGAGTTCAAGCTGAGGACAGAGCAAAGGAAGACCTTTGTGGAACTCAT
GAAGAGAGGAGACCTTCCCGTTTGGCTGGCCTATCAAGTAGCATCTGCCGGAATAACTTACACAGATAGAAGATGG
TGCTTTGATGGCACTACCAACAACACCATAATGGAAGACAGTGTACCAGCAGAGGTGTGGACCAAGTATGGAGAG
AAGAGAGTGCTTAAACCGAGGTGGATGGATGCTAGGGTCTGTTCAGATCATGCGGCTCTGAAGTCGTTCAAAGAAT
TTGCCGCTGGGAAGAGAGGAGCGGCTTTGGGAGTAATGGATGCCCTGGGAACATTGCCAGGACACATGACAGAGA
GGTTTCAGGAAGCCATTGACAATCTCGCTGTGCTCATGCGAGCAGAGACTGGAAGTAGGCCCTACAAAGCAGCGG
CAGCTCAACTGCCGGAGACCTTAGAGACTATCATGCTTCTGGGCTTATTGGGAACAGTTTCGCTAGGAATCTTCTTT
GTCTTGATGCGGAACAAGGGCATCGGGAAGATGGGCTTCGGAATGGTAACCCTTGGGGCCAGCGCATGGCTCATGT
GGCTTTCGGAAATTGAACCAGCCAGAATCGCATGTGTCCTCATTGTCGTGTTTTTGTTGCTGGTGGTGCTCATACCC
GAGCCAGAGAAGCAAAGATCTCCCCAGGATAATCAAATGGCAATCATCATCATGGTGGCAGTTGGCCTTCTGGGTT
TGATAACTGCAAATGAACTCGGATGGCTGGAAAGGACAAAAAGTGATATAGCTCATCTAATGGGAAGGAAAGAAG
AGGGGACAACCATGGGATTCTCAATGGATATTGATCTGCGGCCAGCCTCCGCCTGGGCTATTTATGCCGCATTGAC
AACTCTCATCACCCCAGCCGTCCAACATGCGGTAACCACCTCATACAACAACTACTCCCTGATGGCGATGGCCACA
CAAGCTGGAGTGCTGTTTGGCATGGGCAAAGGGATGCCATTTTATGCATGGGACTTTGGAGTCCCGCTGCTAATGA
TGGGTTGCTACTCACAATTAACACCCCTGACCCTGATAGTGGCCATCATTCTGCTTGTGGCACACTACATGTACTTG
ATCCCAGGTTTGCAGGCAGCAGCAGCgCGTGCTGCCCAGAAGAGGACAGCAGCTGGCATCATGAAGAATCCCGTTG
TGGATGGAATAGTGGTGACTGACATTGACACAATGACAATTGACCCCCAAGTGGAGAAGAAGATGGGACAAGTGT
TACTCATAGCAGTAGCTGTCTCCAGTGCTGTGCTACTGCGGACCGCTTGGGGATGGGGGGAGGCTGGGGCTCTGAT
CACAGCAGCAACCTCCACCTTATGGGAAGGCTCTCCAAACAAGTACTGGAACTCCTCCACAGCCACCTCACTGTGC
AACATCTTTAGAGGAAGTTATTTGGCAGGGGCTTCCCTTATTTACAC

DAK-III

GTACTGGAACTCCTCCACAGCCACCTCACTGTGCAACATCTTTAGAGGAAGTTATTTGGCAGGGGCTTCCCTTATTT
ACACAGTGACAAGAAATGCCGGTCTGGTTAAGAGACGTGGAGGTGGAACGGGAGAGACCCTGGGAGAGAAGTGG
AAAGCCCGCCTGAACCAGATGTCGGCCTTGGAGTTCTACTCTTACAAAAAGTCAGGCATCACCGAAGTGTGTAGGG
AGGAGGCGCGCCGCGCCCTCAAGGATGGAGTGGCCACAGGAGGACATGCTGTATCCCGGGGAAGCGCAAAGCTTA
GATGGTTGGTAGAGAGAGGATACCTGCAGCCCCATGGAAAGGTTGTTGACCTTGGATGTGGCAGAGGAGGCTGGA
GTTATTACGCTGCCACCATCCGTAAAGTGCAGGAGGTCAGAGGATACACAAAGGGAGGTCCTGGCCATGAAGAGC
CCATGCTGGTGCAAAGCTATGGGTGGAACATAGTTCGCCTCAAGAGTGGAGTGGACGTCTTCCACATGGCGGCTGA
ACCGTGTGACACTCTGCTGTGTGACATAGGCGAGTCATCATCCAGTCCTGAAGTGGAAGAGACGCGAACACTCAGA
GTGCTCTCCATGGTGGGAGACTGGCTTGAAAAAAGACCAGGGGCCTTCTGCATAAAGGTGCTGTGCCCATACACCA
GCACAATGATGGAGACCATGGAGCGACTGCAACGTAGGCATGGGGGAGGATTAGTCAGAGTGCCATTGTCCCGCA
ACTCTACACATGAGATGTATTGGGTCTCTGGAGCCAAAAGTAACATCATAAAGAGTGTGTCCACCACAAGTCAGCT
CCTCTTGGGACGCATGGAAGGGCCTAGGAGGCCAGTGAAATATGAGGAGGATGTGAACCTCGGCTCAGGCACACG
AGCTGTGGCAAGCTGCGCTGAGGCTCCCAACATGAAGATCATTGGTAGGCGCATTGAGAGAATCCGCAATGAACA
TGCAGAGACATGGTTCTTTGATGAAAACCACCCATACAGGACATGGGCCTACCATGGGAGCTACGAAGCCCCCACG
CAGGGGTCAGCGTCATCCCTCGTGAACGGGGTTGTTAGACTCTTGTCAAAGCCCTGGGATGTGGTGACTGGAGTTA
CAGGAATAGCTATGACTGACACCACGCCATACGGCCAACAAAGAGTCTTCAAGGAAAAAGTGGACACCAGGGTGC
CAGATCCCCAAGAAGGCACTCGCCAAGTGATGAACATGGTATCGTCTTGGTTATGGAAGGAGCTGGGAAAACGCA
AGCGGCCACGTGTCTGCACCAAAGAAGAGTTCATTAATAAGGTGCGCAGCAATGCAGCACTGGGAGCAATATTTG
AAGAGGAAAAAGAATGGAAGACAGCTGTAGAAGCTGTGAATGATCCAAGATTTTGGGCTCTAGTGGACAAGGAAA
GAGAACACCACCTGAGAGGAGAGTGTCATAGCTGTGTGTACAACATGATGGGCAAAAGAGAAAAGAAGCAAGGA
GAATTCGGGAAAGCAAAAGGCAGCCGTGCAATCTGGTACATGTGGTTGGGAGCCAGATTTTTGGAGTTTGAAGCTC
TTGGGTTCTTGAACGAGGACCACTGGATGGGGAGAGAAAACTCAGGAGGTGGCGTTGAAGGGCTAGGACTGCAAA
GGCTTGGATATATCCTAGAAGAAATGAACCGGGCACCAGGAGGAAAGATGTATGCAGATGACACTGCTGGCTGGG
ACACCCGCATTAGCAAGTTTGATCTAGAGAATGAAGCCCTGATCACTAACCAGATGGAAGAAGGGCACAGAGCTC
TGGCGTTGGCCGTGATTAAATACACATACCAAAACAAAGTGGTGAAGGTTCTCAGACCAGCTGAAGGAGGGAAAA
CAGTCATGGACATCATCTCAAGACAAGACCAGAGAGGGAGCGGACAAGTTGTTACTTATGCTCTCAACACATTCAC
CAACCTGGTGGTGCAGCTTATCCGGAACATGGAGGCTGAGGAAGTGCTAGAGATGCATGATCTGTGGTTGTTGAGG
AAGCCAGAGAAAGTGACCAGATGGTTGCAGAGCAATGGATGGGACAGACTCAAACGGATGGCAGTTAGTGGAGAT
GACTGCGTTGTAAAGCCAATTGATGATAGGTTTGCACATGCCCTCAGGTTCTTGAATGACATGGGAAAGGTTAGGA
AAGACACACAGGAATGGAAACCCTCGACTGGATGGAGCAATTGGGAAGAGGTCCCGTTCTGTTCTCACCACTTCAA
CAAGCTGCACCTCAAGGACGGGAGATCCATTGTGGTCCCCTGCCGCCACCAAGATGAACTGATTGGCCGAGCCCGT
GTCTCACCAGGGGCAGGATGGAGCATCCGGGAGACTGCTTGTCTTGCAAAATCATATGCACAGATGTGGCAGCTTC
TTTATTTCCACAGAAGAGACCTCCGACTGATGGCCAATGCCATTTGTTCGGCTGTGCCAATTGACTGGGTACCAACT

GGGAGAACCACCTGGTCAATCCATGGAAAGGGAGAATGGATGACTACTGAGGACATGCTCATGGTGTGGAATAGA
GTGTGGATTGAGGAGAACGACCACATGGAGGACAAGACCCCTGTAACAAAATGGACAGACATTCCCTATTTGGGA
AAAAGGGAGGACTTATGGTGTGGATCCCTTATAGGGCATAGACCTCGCACCACTTGGGCTGAGAACATCAAAGAC
ACAGTCAACATGGTGCGTAGGATCATAGGTGATGAAGAAAAGTTCATGGACTACCTATCCACCCAAGTACGCTACT
TGGGTGAGGAAGGGTCCACACCTGGAGTGCTGTAAGCATCAATTTCAATGTTGTCAGGCCTGCTAGTCAGCCACAG
TTTGGGGAAAGCTGTGCAGCCTGTAACCCCCCCAGGAGAAGCTGGGAAACCAAGCTCATAGTCAGGCCGATAACG
CCATGGCACGGAAAAAGCCATGCTGCCTGTGAGCCCCTCAGAGGACACTGAGTCAAAAAACCCCACGCGCTTGGA
AGCGCAGGATGGGAAAAGAAGGTGGCGACCTTCCCCACCCTTTAATCTGGGGCCTGAACTGGAGATTAGCTGTGA
ATCTCCAGCAGAAGGACTAGTGGTTAGAGGAGACCCCCCGGAAAACGCAAAACAGCATATTGACGCTGGGAAAGA
CCAGAGACTCCATGAGTTTCCACCACGCTGGCCGCCAGGCACAGATCGCCGAACAGCGGCGGCCGGTGTGGGGAA
ATCCATGGGTCTGGCCGGCATGGTCCCAGCCTCCTCGCTGGCGCCGGCTGGGCAACATTCCGAGGGGACCGTCCCC
TCGGTAATGGCGAATGGGACTCGCGACAGACATGATAAGATACATTGATGAGTTTGGACAAACCACAACTAGAAT
GCAGTGAAAAAAATGCTTTATTTGTGAAATTAAGCGCTGGCATTGACCCTGAG

MART-II

GAAACATGTGGAACAAGAGGACCATCTCTGAGATCAACCACTGCAAGCGGAAGGGTGATCGAGGAATGGTGCTGC
AGGGAGTGCACAATGCCCCCACTGTCGTTCTGGGCTAAAGATGGCTGTTGGTATGGAATGGAGATAAGGCCCAGG
AAAGAACCAGAAAGCAACTTAGTAAGGTCAATGGTGACTGCAGGATCAACTGATCACATGGATCACTTCTCCCTTG
GAGTGCTTGTGATTCTGCTCATGGTGCAGGAAGGGCTGAAGAAGAGAATGACCACAAAGATCATCATAAGCACAT
CAATGGCAGTGCTGGTAGCTATGATCCTGGGAGGATTTTCAATGAGTGACCTGGCTAAGCTTGCAATTTTGATGGG
TGCCACCTTCGCGGAAATGAACACTGGAGGAGATGTAGCTCATCTGGCGCTGACAGCGGCATTCAAAGTCAGACCA
GCGTTGCTGGTATCTTTCATCTTCAGAGCTAATTGGACACCCCGTGAAAGCATGCTGCTGGCCTTGGCCTCGTGTCT
TTTGCAAACTGCGATCTCTGCCTTGGAAGGCGACCTGATGGTTCTCATCAATGGTTTTGCTTTGGCCTGGTTGGCAA
TACGAGCGATGGTTGTTCCACGCACTGACAACATCACCTTGGCAATCCTGGCTGCTCTGACACCACTGGCCCGGGG
CACACTGCTTGTGGCGTGGAGAGCAGGCCTTGCTACTTGCGGGGGGTTTATGCTCCTCTCTCTGAAGGGAAAAGGC
AGTGTGAAGAAGAACTTACCATTTGTCATGGCCCTGGGACTAACCGCTGTGAGGCTGGTCGACCCCATCAACGTGG
TGGGACTGCTGTTGCTCACAAGGAGTGGGAAGCGGAGCTGGCCCCCTAGCGAAGTACTCACAGCTGTTGGCCTGAT
ATGTGCATTGGCTGGAGGGTTCGCCAAGGCAGATATAGAGATGGCTGGGCCCATGGCCGCGGTCGGTCTGCTAATT
GTCAGTTACGTGGTCTCAGGAAAGAGTGTGGACATGTACATTGAAAGAGCAGGTGACATCACATGGGAAAAAGAT
GCGGAAGTCACTGGAAACAGTCCCCGGCTCGACGTGGCGCTAGATGAGAGTGGTGATTTCTCCCTGGTGGAGGATG
ACGGTCCCCCCATGAGAGAGATCATACTCAAGGTGGTCCTGATGACCATCTGTGGCATGAACCCAATAGCCATACC
CTTTGCAGCTGGAGCGTGGTACGTATACGTGAAGACTGGAAAAAGGAGTGGTGCTCTATGGGATGTGCCTGCTCCC
AAGGAAGTAAAAAAGGGGGAGACCACAGATGGAGTGTACAGAGTAATGACTCGTAGACTGCTAGGTTCAACACAA
GTTGGAGTGGGAGTTATGCAAGAGGGGGTCTTTCACACTATGTGGCACGTCACAAAAGGATCCGCGCTGAGAAGC
GGTGAAGGGAGACTTGATCCATACTGGGGAGATGTCAAGCAGGATCTGGTGTCATACTGTGGTCCATGGAAGCTAG
ATGCCGCCTGGGACGGGCACAGCGAGGTGCAGCTCTTGGCCGTGCCCCCCGGAGAGAGAGCGAGGAACATCCAGA
CTCTGCCCGGAATATTTAAGACAAAGGATGGGGACATTGGAGCGGTTGCGCTGGATTACCCAGCAGGAACTTCAGG
ATCTCCAATCCTAGACAAGTGTGGGAGAGTGATAGGACTTTATGGCAATGGGGTCGTGATCAAAAATGGGAGTTAT
GTTAGTGCCATCACCCAAGGGAGGAGGGAGGAAGAGACTCCTGTTGAGTGCTTCGAGCCTTCGATGCTGAAGAAG
AAGCAGCTAACTGTCTTAGACTTGCATCCTGGAGCTGGGAAAACCAGGAGAGTTCTTCCTGAAATAGTCCGTGAAG
CCATAAAAACAAGACTCCGTACTGTGATCTTAGCTCCAACCAGGGTTGTCGCTGCTGAAATGGAGGAAGCCCTTAG
AGGGCTTCCAGTGCGTTATATGACAACAGCAGTCAATGTCACCCACTCTGGAACAGAAATCGTCGACTTAATGTGC
CATGCCACCTTCACTTCACGTCTACTACAGCCAATCAGAGTCCCCAACTATAATCTGTATATTATGGATGAGGCCCA
CTTCACAGATCCCTCAAGTATAGCAGCAAGAGGATACATTTCAACAAGGGTTGAGATGGGCGAGGCGGCTGCCATC
TTCATGACCGCCACGCCACCAGGAACCCGTGACGCATTTCCGGACTCCAACTCACCAATTATGGACACCGAAGTGG
AAGTCCCAGAGAGAGCCTGGAGCTCAGGCTTTGATTGGGTGACGGATCATTCTGGAAAAACAGTTTGGTTTGTTCC
AAGCGTGAGGAACGGCAATGAGATCGCAGCTTGTCTGACAAAGGCTGGAAAACGGGTCATACAGCTCAGCAGAAA
GACTTTTGAGACAGAGTTCCAGAAAACAAAACATCAAGAGTGGGACTTTGTCGTGACAACTGACATTTCAGAGATG
GGCGCCAACTTTAAAGCTGACCGTGTCATAGATTCCAGGAGATGCCTAAAGCCGGTCATACTTGATGGCGAGAGAG
TCATTCTGGCTGGACCCATGCCTGTCACACATGCCAGCGCTGCCCAGAGGAGGGGGCGCATAGGCAGGAATCCCAA
TAAACCTGGAGATGAGTATCTGTATGGAGGTGGGTGCGCAGAGACTGACGAAGACCATGCACACTGGCTTGAAGC
AAGAATGCTCCTTGACAATATTTACCTCCAAGATGGCCTCATAGCCTCGCTCTATCGACCTGAGGCCGACAAAGTA
GCAGCCATTGAGGGAGAGTTCAAGCTTAGGACGGAGCAAAGGAAGACCTTTGTGGAACTCATGAAAAGAGGAGAT
CTTCCTGTTTGGCTGGCCTATCAGGTTGCATCTGCCGGAATAACCTACACAGATAGAAGATGGTGCTTTGATGGCAC
GACCAACAACACCATAATGGAAGACAGTGTGCCGGCAGAGGTGTGGACCAGACACGGAGAGAAAAGAGTGCTCA
AACCGAGGTGGATGGACGCCAGAGTTTGTTCAGATCATGCGGCCCTGAAGTCATTCAAGGAGTTTGCCGCTGGGAA
AAGAGGAGCGGCTTTTGGAGTGATGGAAGCCCTGGGAACACTGCCAGGACACATGACAGAGAGATTCCAGGAAGC
CATTGACAACCTCGCTGTGCTCATGCGGGCAGAGACTGGAAGCAGGCCTTACAAAGCCGCGGCGGCCCAATTGCCG

GAGACCCTAGAGACCATTATGCTTTTGGGGTTGCTGGGAACAGTCTCGCTGGGAATCTTTTTCGTCTTGATGAGGAA
CAAGGGCATAGGGAAGATGGGCTTTGGAATGGTGACTCTTGGGGCCAGCGCATGGCTCATGTGGCTCTCGGAAATT
GAGCCAGCCAGAATTGCATGTGTCCTCATTGTTGTGTTCCTATTGCTGGTGGTGCTCATACCTGAGCCAGAAAAGCA
AAGATCTCCCCAGGACAACCAAATGGCAATCATCATCATGGTAGCAGTAGGTCTTCTGGGCTTGATTACCGCCAAT
GAACTCGGATGGTTGGAGAGAACAAAGAGTGACCTAAGCCATCTAATGGGAAGGAGAGAGGAGGGGGCAACCAT
AGGATTCTCAATGGACATTGACCTGCGGCCAGCCTCAGCTTGGGCCATCTATGCTGCCTTGACAACTTTCATTACCC
CAGCCGTCCAACATGCAGTGACCACTTCATACAACAACTACTCCTTAATGGCGATGGCCACGCAAGCTGGAGTGTT
GTTTGGTATGGGCAAAGGGATGCCATTCTACGCATGGGACTTTGGAGTCCCACTGCTAATGATAGGTTGCTACTCA
CAATTAACACCCCTGACCCTAATAGTGGCCATCATTTTGCTCGTGGCGCACTACATGTACTTGATCCCAGGGCTGCA
GGCAGCAGCTGCGCGTGCTGCCCAGAAGAGAACGGCAGCTGGCATCATGAAGAACCCTGTTGTGGATGGAATAGT
GGTGACTGACATTGACACAATGACAATTGACCCCCAAGTGGAGAAAAAGATGGGACAGGTGCTACTCATAGCAGT
AGCCGTCTCCAGCGCCATACTGTCGCGGACCGCCTGGGGGTGGGGGGAGGCTGGGGCCCTGATCACAGCCGCAAC
TTCCACTTTGTGGGAAGGCTCTCCGAACAAGTACTGGAACTCCTCTACAGCCACTTCACTGTGTAACATTTTTAGGG
GAAGTTACTTGGCTGGAGCTTC
